# Platelet Aggregometry Testing: Molecular Mechanisms, Techniques and Clinical Implications

**DOI:** 10.3390/ijms18081803

**Published:** 2017-08-18

**Authors:** Katalin Koltai, Gabor Kesmarky, Gergely Feher, Antal Tibold, Kalman Toth

**Affiliations:** 1 First Department of Medicine, University of Pecs, Pecs 7624, Hungary; koltai.katalin@pte.hu (K.K.); kesmarky.gabor@pte.hu (G.K.); 2 Hospital of Szigetvar, Szigetvar 7900, Hungary; feher.gergely@pte.hu; 3 Department of Public Health Medicine, University of Pecs, Pecs 7624, Hungary; tibold.antal@pte.hu

**Keywords:** platelet aggregation, aggregometry, platelet function disorders, tailored antiplatelet therapy, aspirin, clopidogrel, thienopyridines

## Abstract

Platelets play a fundamental role in normal hemostasis, while their inherited or acquired dysfunctions are involved in a variety of bleeding disorders or thrombotic events. Several laboratory methodologies or point-of-care testing methods are currently available for clinical and experimental settings. These methods describe different aspects of platelet function based on platelet aggregation, platelet adhesion, the viscoelastic properties during clot formation, the evaluation of thromboxane metabolism or certain flow cytometry techniques. Platelet aggregometry is applied in different clinical settings as monitoring response to antiplatelet therapies, the assessment of perioperative bleeding risk, the diagnosis of inherited bleeding disorders or in transfusion medicine. The rationale for platelet function-driven antiplatelet therapy was based on the result of several studies on patients undergoing percutaneous coronary intervention (PCI), where an association between high platelet reactivity despite P2Y12 inhibition and ischemic events as stent thrombosis or cardiovascular death was found. However, recent large scale randomized, controlled trials have consistently failed to demonstrate a benefit of personalised antiplatelet therapy based on platelet function testing.

## 1. Introduction

An initial adhesion of platelets to the site of injury is mediated by the binding of collagen exposed at the damaged vessel wall to platelet surface glycoprotein VI (GPVI) and integrin α2β1, and by the binding of von Willebrand factor (vWF) to the platelet surface glycoprotein Ib(GP1b)-IX-V complex. GPVI is a 58 kD platelet membrane glycoprotein receptor for collagen that plays a pivotal role in the collagen–induced activation of platelets. Vessel wall injury and exposure of the subendothelial matrix to blood flow leads to adhesion of platelets. Collagen fibers have a highly thrombogenic property with collagen types I, III and IV being the most common forms to be found in blood vessels. The initial adhesion of platelets is followed by platelet-to-platelet adhesion. Different agonists are present at the site of vessel wall injury that play a role after adhesion in platelet activation such as adenosine diphosphate (ADP) and collagen. Platelets are activated by agonists binding to specific receptors that are presented on the platelet surface membrane. Platelet activation leads to a series of events that eventually increases the intracytoplasmatic concentration of calcium ions through release from intracellular stores and calcium influx from the plasma [1].

For normal ADP induced platelet aggregation a co-activation of P2Y12 and P2Y1 receptors is necessary, while the inhibition of either is sufficient to inhibit it. P2X1 is a ligand gated ion channel that is responsible for a rapid phase of adenine-nucleotid evoked Ca^2+^ influx through a kation channel. 

G-protein coupled receptors as adenosine diphosphate (ADP) thromboxane A2 (TXA2) and thrombin receptors activate phospholipase Cβ (PLCβ). Collagen receptor glycoprotein VI (GPVI) is acting via the non-receptor tyrosine kinase pathways and activates phospholypase Cγ (PLCγ) [2]. These processes produce changes within the platelet that lead to the transformation of GPIIb/IIIa complexes (also known as integrin αIIbβ3) that enables them to bind plasma fibrinogen. Activation of PLCβ or PLCγ initiates the production of diacylglycerol (DAG) and inositol trisphosphate (IP3). DAG helps calcium influx, IP3 mobilizes intracellular calcium stores. Certain agonists as adenosine triphosphate (ATP) also directly induce calcium influx by binding to the ligand-gated non-selective kation channel receptor P2X1 [3].

Platelet responses may be reversible or irreversible. Reversible platelet responses include adhesion, shape change and reversible aggregation. Irreversible platelet response includes the release reaction and secondary irreversible aggregation.

Increased free calcium concentration within the platelet causes certain structural and functional alterations. The originally discoid shape of the platelet changes to a sphere with long membrane projections. Platelet granules become centralized and they empty their content into the canalicular system. From there they are discharged into the plasma, a process called release reaction. Membrane phopholipase A2 activity is stimulated by increased calcium concentration within the platelets, which leads to the liberation of arachidonic acid (AA) from membrane phospholipids. Cyclooxygenase-1 (COX-1) converts arachidonic acid to prostaglandin H2 (PGH2), an intermediate product that is further metabolized by thromboxane synthase into TXA2 [4]. TXA2 is a strong activator of platelets.

Shape change reaction of platelets is mediated by the platelet cytoskeleton, a network of microtubules and actin filaments. Shape change causes basical changes in the organisation of the cytoskeleton, resulting in the polymerisation of actin and myosin light chain phosphorylation [5,6,7,8]; it results in the transformation of platelets from biconcave disks to spread cells. During this process, platelets extend long, spiky membrane projections resulting in a dramatic increase in the platelet surface area, enabling platelets to interact with each other and aggregate. Platelet shape change reaction depends on the provoking stimulus and varies in time.

The role of GPIIb/IIIa membrane protein complex is fundamental in platelet aggregation. It is a receptor for fibrinogen and von Willebrand factor. It is presented in high density on the plasma membrane and on the α-granules of platelets [9]. In resting platelets it stays in its inactive form. The complex is formed via calcium-dependent association of GPIIb and GPIIIa, which is a necessary step in normal platelet aggregation. Platelet activation by ADP and by most other agonists causing platelet activation induce conformational changes in GPIIb/IIIa that transform it into its fibrinogen binding form [10,11]. The receptor-bound fibrinogen connects two GPIIb/IIIa molecules on nearby platelets. This process is the final common pathway of agonist-induced platelet aggregation and platelet aggregation under low shear situations. In the case of high shear induced platelet aggregation, vWF plays a role instead of fibrinogen in bridging GPIIb/IIIa receptors on adjacent platelets [12]. The GPIIb/IIIa receptor is a target for several antiplatelet agents as abciximab, eptifibatide and tirofiban.

Junctional adhesion molecules (JAMs), signaling lymphocyte activation molecule (SLAM) family proteins, and CD40 ligand [13,14] have also been suspected to have a potential to bridge and bind platelets to one another and stabilize thrombi, although their role is not yet fully elucidated.

Agonist-initiated signaling in platelets is closely regulated in order to avoid excessive responses to injury. On circulating platelets, GPIIb/IIIa in a resting conformation is incapable of binding its ligand, fibrinogen. Agonist induced platelet activation converts the integrin complex in a high-affinity state through a cascade of signaling events called inside-out signaling that culminate in the exposure of high-affinity binding sites on GPIIb/IIIa. After the activation of the platelet, GPIIb/IIIa, which is the receptor with the highest copy number per platelet, stabilizes thrombus formation by providing agonist-independent "outside-in" signals what results in platelet adhesion, spreading and clot retraction. A tightly regulated mechanism of agonist-dependent integrin modulation prevents unwanted activation and signaling. Similarly, outside-in signaling is also elaborately regulated by both positive and negative regulators. Junctional adhesion molecule A (JAM-A) is rapidly phosphorilated during platelet activation by physiological agonists in a protein kinase C-dependent manner. It normally limits platelet accumulation by inhibiting integrin outside-in signaling thus preventing premature platelet activation. A loss of JAM-A results in a prothrombotic phenotype [15].

There are numerous feedback amplification loops that help in the build-up and consolidation of the hemostatic plug by recruiting other platelets at the site of vascular injury. Activated platelets synthesize de novo TXA2, and release platelet agonist substances as ADP and serotonin from the α-granules. ADP stimulates P2Y purinoceptor 1 (P2Y1) and P2Y12, serotonin stimulates 5-hydroxytriptamine 2A receptors (5HT2A). The thromboxane prostanoid (TP) receptor is stimulated by the binding of fibrinogen (or at high shear of vWF) to the activated conformation of GPIIb/IIIa [16].

## 2. Techniques of Platelet Aggregometry

### 2.1. Thromboxane A_2_ Metabolites

TXA2 is the major product of arachidonic acid metabolism in platelets. It is synthesized and released from platelets at the site of vascular injury, amplifiying platelet activation [17]. Arachidonic acid is derived from membrane phospholipids through phospholipase A2 (PLA2). It is transformed by cyclooxigenase-1 (COX-1) into prostaglandin G2 and prostaglandin H2, then transformed by thromboxane synthase into TXA_2_, a potent vasoconstrictor. TXA_2_ is transformed by hydrolysis into biologically inactive and stable thromboxane B_2_ (TXB_2_) [18]. TXB_2_ is transformed into two major metabolites: 2,3-dinor-TXB2 by β-oxidation and in 11-dehydro-TXB2 by dehydration. 11-dehydro-TXB_2_ is excreted in urine and its urinary level reflects the total capacity of platelets to synthesize TXA_2_ [19]. Aspirin effect on COX-1 decreases the concentration of urinary 11-dehydro-TXB_2_ [20,21]. Assessment of TXA2 metabolites reflects the activation of platelets and the quantity of TXA_2_ production, however, about 30% of 11-dehydro-TXB_2_ originates from sources other than the platelets [22,23,24]. TXA_2_ metabolites measured by ligand binding assays are used to monitor antiplatelet therapy, as they reflect the in vivo effect of aspirin on platelets [25,26,27,28]. Enzyme-linked immunoassays (ELISA) are nowadays applied [29,30] rather than radioimmunassay (RIA) or immunoradiometric assays (IRMA) [31,32,33].

### 2.2. Optical Aggregometry

The historical “gold standard” is turbidometric platelet aggregometry, which measures platelet aggregation in PRP (platelet-rich plasma) [25,34]. The method is based on the detection of difference in light transmission by a photometer after adding a platelet agonist to platelet rich plasma. Aggregation measurements provide an aggregation index curve describing the light transmission intensity changes of the PRP samples. Samples can be exposed to a wide range of agonists, which can give an insight into different pathways of platelet activation/aggregation. Maximal extent of aggregation expressed as percentages, lag phase and slope of the curve are routinely measured parameters.

Optical aggregometry has been widely used for monitoring antiplatelet therapies. For this indication the most commonly used agonists are ADP, AA, collagen, and epinephrine. Optical aggregometry can be used to monitor acetyl-salicylic acid (ASA), thienopyridine and platelet GP IIb/IIIa inhibitor therapy. Its disadvantages include the large sample volumes required, long processing times and complex sample preparation [26,27,35]. Certain clinical conditions as hemolysis, low platelet count or severe hyperlipidemia may also influence the result of the test.

Optical aggregometry is a widely used tool in the diagnosis of congenital and acquired platelet function disorders. Further agonists as ristocetin, thrombin receptor activating peptide (TRAP), thromboxane A2 mimetic U46619, calcium ionophore A23187 can be used for this indication. Ristocetin is an antibiotic that helps the binding of vWF to the glycoprotein Ib/IX/V complex. Both normal vWF and glycoprotein Ib/IX/V complex must be present for a normal result. Ristocetin-induced platelet aggregation abnormalities are usually associated with von Willebrand factor deficiency or dysfunction. The test can be used in the diagnostics of von Willebrand disease as well as platelet function disorders as Bernard-Soulier syndrome [36,37].

Drugs that inhibit COX-1 reduce aggregation with lower concentrations of collagen and result in a missing secondary aggregation using epinephrine. Aggregation is absent or significantly reduced using AA but normal with TXA2 analogue U46619.

ADP receptor blocker drugs (e.g., thyenopyridines) markedly reduce ADP induced platelet aggregation and cause abnormal aggregation with multiple agonists. Similar alterations can be seen in the case of defects of the platelet P2Y12 receptor.

If aggregation is only present with ristocetin, or significantly impaired with all agonists except for ristocetin with a normal platelet count, normal platelet size or macrothrombocytopenia, Glantzmann thrombasthenia due to inherited or acquired abnormalities of integrin αIIbβ3 can be suspected in the background.

Aggregation reduced with the use of high concentrations of ristocetin with a normal platelet count can be a sign of von Willebrand disease. However, type 2B or platelet type vWF disease due to a defect of platelet-vWF interactions is characterized by abnormally increased aggregation using low concentrations of ristocetin, with possible thrombocytopenia and platelet clumping.

An absent aggregation using high concentrations of ristocetin with thrombocytopenia and very large platelets can refer to Bernard-Soulier syndrome caused by inherited or acquired abnormalities of GP-Ib-IX-V. In this case, vWF deficiency should be excluded.

If aggregation is markedly reduced with collagen but normal with other agonists, a platelet collagen receptor defect involving glycoprotein VI or α2β1 may be in the background. In the case of an abnormality only with epinephrine, Quebec platelet disorder should be considered, especially if there is a history of delayed bleeding [38].

Monitoring antiplatelet therapies (e.g., ASA and thienopyridine) by using light transmission aggregometry (LTA) proved to be predictive concerning major adverse cardiovascular events (MACE) in high-risk cardiovascular patients. Nonresponsiveness to clopidogrel defined with ADP induced platelet aggregation proved to be a strong independent predictor of stent thrombosis in patients receiving sirolimus- or paclitaxel-eluting stents. ADP and arachinonic acid induced platelet aggregation have been associated with the development of ischemic events. 

A number of previous studies found a lack of reproducibility of assessment of aspirin responsiveness by LTA [39]. In a study of 207 patients treated with aspirin, a very good short-term intra-individual reproducibility of LTA assessment of platelet reactivity was found, while long-term reproducibility was poor, indicating that in the long-term perspective the antiplatelet ASA effectivity may be influenced by additional variables [40]. On the other hand, the method was found highly reproducible and concordant in healthy individuals and in patients with mild platelet function disorders [41]. In a recent study to assess sources of variability in platelet function tests in normal subjects, a flavonoid-rich diet has affected LTA results in healthy subjects [42].

As different types and doses of agonists as well as different cut-off values have been used in different centers, study and clinical results were often not fully comparable. For this reason, efforts have been made to standardize the method [43,44,45] as well as specific guidelines have been published [38,46,47,48]. However, there are several methodological differences between the different guidelines as it is reflected in Table 1.

### 2.3. Platelet Function Analyzer

The platelet function analyzer PFA-100 and Innovance PFA-200 (Siemens, Munich, Germany) measures in vitro the cessation of high-shear blood flow by the platelet plug. It is a simple, rapid, point-of-care whole blood method that requires low sample volumes and no sample preparation. Its disadvantages are that it is dependent on von Willebrand factor and hematocrit levels and that it requires pipetting. Two different cartridges-collagen plus ADP (CAPD) and collagen plus epinephrine (CEPI) are applied in the PFA-100 system. Citrated whole blood flows through a capillary at a high shear rate within the cartridges that ends in a collagen coated membrane with a 147 μm aperture filled with ADP or EPI. The time until clot buildup occludes the aperture is called closure time (CT).

The method has been used for monitoring the effect of acetyl-salicylic acid therapy. A short CEPI CT could be indicative of high residual platelet reactivity in patients on aspirin therapy [49,50]. Shortened CEPI closure time in acute myocardial infarction patients is associated with higher prevalence of MACE [51,52,53,54]. This method (combined with optical aggregometry) has proved to be useful in the detection of residual platelet activity [35]. However, in other studies agreement between PFA results and other platelet aggregometry methods was poor [55].

PFA-100 is not recommended for monitoring the effect of thienopyridines [35]. However, the recently available INNOVANCE PFA P2Y cartridge proved to be sensitive to P2Y_12_ inhibition and was comparable to other currently available platelet function tests [56].

Although the PFA-100 CT is abnormal in some forms of platelet disorders [57,58], the test did not prove to have sufficient sensitivity or specificity to be used as a screening tool for platelet disorders. Thus, the PFA-100 closure time should be considered optional in the evaluation of platelet disorders and function, and its use in therapeutic monitoring of platelet function is recommended to be restricted to research studies and prospective clinical trials [58,59].

PFA-100 may provide useful information on patients scheduled for surgery for postoperative blood transfusion management [60,61]. In cardiac surgery patients PFA could predict intra- and postoperative blood loss [62,63,64,65]. Prolonged CADP CTs have been proved to be independent risk factors for postpartum hemorrhage (PPH) severity [66,67].

### 2.4. Impedance Aggregometry

Impedance aggregometry measures the change in electrical impedance between two electrodes when platelet aggregation is induced by an agonist. The principle of the method is similar to that of optical aggregometry except that it can be done in whole blood, thus obviating the need for preparation of a platelet suspension. Platelets aggregate to platelets fixed to the electrodes what causes an increase in electrical impedance. Platelet aggregation is assessed by detecting the increase in electrical impedance recorded in Ohms [68]. Impedance aggregometry can also be performed in thrombocytopenic patients [69].

Impedance aggregometry assesses platelet function under more physiological conditions as it is performed in whole blood, thus enabling other blood elements to influence platelet aggregation. Also, it takes place on a solid surface resembling the physiological process of platelet adhesion and aggregation.

The Multiple Electrode Aggregometry (MEA) is based on impedance aggregometry, however, it can be used as a point-of -care device [70]. It has 5 channels for simultaneous measurement of different samples or agonists. Each cuvette contains two pairs of sensor electrodes as an integrated quality control. Platelet aggregation is simultaneously measured in duplicate by using each sensor unit separately. Pipetting is automated. MEA can be used for monitoring antiplatelet therapies and detect high on-treatment platelet reactivity [71,72,73,74]. It is also suitable to identify patients with a bleeding diathesis.

MEA can be used to identify patients at risk of blood loss pre- or intraoperatively [75,76]. The MEA ADP test in patients on thienopyridine therapy who undergo cardiac surgery was associated with postoperative bleeding and the need of platelet transfusion, and proved to be a useful method to predict postoperative bleeding risk [77,78,79]. According to several studies MEA can be used to identify patients at high risk of bleeding [80].

### 2.5. Thromboelastography, Thromboelastogram Platelet Mapping System and ROTEM Platelet Test

Thromboelastography (TEG) and thromboelastometry measure viscoelastic changes of the entire clotting process. Different tests are available for the extrinsic and for the intrinsic pathway. By the selective activation of the extrinsic pathway it is possible to evaluate the role of platelets in clot formation. The thromboelastogram platelet mapping system and thromboelastometry platelet test measure platelet contribution to clot strength more specifically. Both tests involve the global functional roles of platelets in hemostasis: thrombin generation, clotting, clot retraction and fibrinolytic activation [81].

The methods most widely used based on these principles are thromboelastography (TEG Platelet Mapping System; Haemoscope, Braintree, MA, USA), thromboelastometry (ROTEM; TEM Int, Munich, Germany), and Sonoclot analysis (Sonoclot Signature; Sienco, Arvada, CO, USA). TEG and ROTEM include a rotating system with a pin suspended by a torsion wire. The Sonoclot device is installed with a pin that is moved up and down at an ultrasonic rate [82]. The test is started by adding appropriate reagents to whole blood samples. Changes in elasticity are measured and displayed at all stages of the developing and resolving clot.

These methods can be used in several different clinical settings to predict the risk of excessive postoperative bleeding and the need for blood products [83,84,85,86,87,88,89,90,91,92,93].

The TEG platelet mapping system is a modification of the original TEG. It is a point-of-care method that is apt to monitor all types of antiplatelet therapies [94]. It provides information about platelets through four different whole blood tests. A kaolin activated sample produces a strong thrombin response cleaving all available fibrinogen, demonstrating the potential for maximum clot strength. One aliquot containing only Activator F blocking all thrombin demonstrates the clot strength coming from fibrin. The 3rd and 4th assays also block all thrombin and activate platelets at either the ADP receptor or at the thromboxane A2 receptor. The degree of inhibition is calculated using the patient’s full hemostatic potential as the baseline.

The ROTEM Platelet System is a new module that can be added to the ROTEM. The method is based on impedance aggregometry in whole blood. It provides information concerning platelet function and aggregation, as well as information about the effect of platelet function influencing drugs. However, being a relatively new method, only few data are available on its usefulness in clinical practice [95].

### 2.6. Flow Cytometry Methods

Flow cytometry (FC) analysis of platelets include various assays for several purposes, i.e., the assessment of platelet activation state, examination of thrombopoiesis, diagnosis of platelet function disorders, and monitoring antiplatelet therapy [96,97].

FC measures antibodies conjugated to fluorescent dyes that are capable of binding specific proteins either on cell membranes or inside cells, thus displaying their presence. A light source excites the fluorescent molecules of platelet-bound dyes to a higher energy state. The dyes emit light at different wavelengths when they return to a resting state. Double labeling means that a specific secondary antibody can be coupled to a fluorochrome, which recognizes the primary antibody [98]. Double labeling is used to identify platelets, platelet microparticles, or mixed aggregates [99,100,101].

Several inherited and acquired platelet function disorders can be diagnosed using FC as Bernard–Soulier syndrome, Glanzmann’s thrombasthenia or heparin-induced thrombocytopenia (HIT). It can be used in the case of severe thrombocytopenia as well [24]. It is also used in transfusion medicine to examine the activation state of stored platelets. FC enables assessment of the activation state of platelets by measuring the expression of phosphatidylserine on activated platelet membranes [102]. 

The most thoroughly studied types of activation dependent monoclonal antibodies are those that are either directed against conformational changes of GPIIb/IIIa or against granule membrane proteins. Monoclonal antibody PAC-1 is directed against the fibrinogen binding site exposed by a conformational change in GPIIb/IIIa due to platelet activation. Thus, PAC-1 binds only to activated platelets. Another commonly used platelet activation related surface marker is directed against platelet surface P selectin (CD62P). As P selectin is expressed on the platelet surface membrane only after α-degranulation, P-selectin specific monoclonal antibodies bind only to degranulated platelets. However, circulating degranulated platelets were found to rapidly lose their P-selectin in vivo, which can limit the use of this method.

Vasodilator-stimulated phosphoprotein (VASP) phosphorylation measures activation-dependent platelet signaling. Its advantages include small required sample volumes, the use of whole blood, stability (allowing samples to be shipped to a remote laboratory) and dependency on the P2Y12 receptor, which is the site of action for clopidogrel. Its disadvantages are that it requires complex sample preparation and experienced technicians [103,104].

### 2.7. VerifyNow

VerifyNow (Accriva Diagnostics, San Diego, CA, USA) is a point-of care device that measures platelet aggregation by turbidimetric–based optical detection in anticoagulated whole blood. Fibrinogen-coated beads augment platelet aggregation; platelets aggregate on the surface of the beads according to the quantity of activated GP IIB/IIIA receptors. Aspirin Test using AA as agonist can be used to investigate aspirin effect. Platelet Reactivity Unit (PRU) Test uses ADP as agonist and PGE_1_ as suppressor of intracellular free calcium to evaluate clopidogrel effect. A second channel investigates thrombin receptor activating peptide (TRAP-) induced platelet aggregation that serves as baseline. The method is fast and simple and uses only a small sample volume. No pipetting is required.

### 2.8. Impact-R

The Impact R analyser (DiaMed, Cressier, Switzerland) determines shear-induced platelet aggregation with a cone-and plate technology. The device tests platelet adhesion and aggregation in anti-coagulated whole blood under arterial flow conditions. Upon application of a blood sample into a polystyrene well, plasma proteins immediately adhere to the well surface resulting in platelet adhesion and aggregation. An image analyser quantifies adhered platelets. Results are expressed as percentage of well surface covered by aggregates as an index of adhesion and average aggregate size as an index of aggregation. The instrument has a research version with adjustable shear rate and a clinical version. The advantage of the method is simplicity, no sample preparation and low sample volumes. However, it is not a real point-of care method as it requires pipetting.

### 2.9. Global Thrombosis Test

The global thrombosis test (GTT) (Montrose Diagnostics Ltd., London, UK) is a novel method based on platelet activation due to high shear stress using native non-anticoagulated whole blood [105]. It is a fast point-of-care test that gives an insight into the thrombotic status of the patient. Its clinical role is under evaluation.

### 2.10. Plateletworks

Plateletworks (Helena Laboratories, Beaumont, TX, USA) is based on GP IIb/IIIa dependent platelet aggregation. Platelet count is compared in samples with and without agonists (ADP or AA). The method requires minimal sample preparation, and it is performed from whole blood. Its disadvantage is that samples have to be measured within a few minutes after blood draw. This feature limits its use and the method has been associated with clinical outcomes only in a few studies.

### 2.11. In Vivo Monitoring of Platelet Aggregation

As most platelet function tests describe platelet aggregation under controlled experimental conditions, they may not reflect the complex process of in vivo thrombus formation. Although intravital microscopy has been used to investigate thrombus formation as early as the end of the 19th century [106], modern imaging techniques have vastly expanded the potential of experimental in vivo monitoring methods. Different intravital video systems have been developed to investigate the kinetics of in vivo platelet adhesion and aggregation in real time [107]. These methods usually involve an injury to vessel walls, for example by micropuncture, chemical or electrical stimulation, laser or photochemical injury [108,109,110,111]. In models based on micropuncture or chemical stimulation platelet adhesion occurs at sites of endothelial denudation, while other techniques provoke platelet adhesion without overt endothelial injury [112].

## 3. Platelet Aggregometry Testing to Monitor Antiplatelet Therapy

Several studies have shown that the result of platelet aggregometry is associated with clinical outcomes in patients undergoing PCI (Table 2).

It has also been proved by numerous trials that different methods of platelet aggregometry may forecast bleeding risk in distinct groups of patients (Table 3).

In patients undergoing endovascular neurointerventional procedures clopidogrel hyper-responsiveness was associated with hemorrhage while clopidogrel resistance was associated with thromboembolism [133,134,135]. However, the threshold values of hyperresponsiveness to antiplatelet drugs are even less settled than in the case of antiplatelet resistance.

Several earlier, smaller studies implied that tailored antiplatelet therapy for clopidogrel low- or non-responders may be effective in reducing the risk of ischemic events. Monitoring-driven changes in therapy included elevating clopidogrel dosage or switching to another thienopyridin drug or adding a non-thienopyridine antiplatelet agent (e.g., GP II b/IIIa receptor inhibitors or cilostazol) [136,137,138,139,140,141,142,143,144].

Later large, randomized, controlled trials were performed on cardiological patients regarding the potential benefits of platelet aggregometry/platelet function testing that failed to show similar positive effects.

The Gauging Responsiveness with a VerifyNow Assay-Impact on Thrombosis and Safety (GRAVITAS) trial was a randomized, double-blind multicenter trial that compared the use of standard dose (75 mg daily) and high dose (600 mg loading dose, then 150 mg daily) clopidogrel treatment in patients undergoing percutaneous coronary intervention with high on-treatment platelet reactivity following percutaneous coronary intervention. No significant difference was found between the standard and high-dose clopidogrel group in the primary end points (cardiovascular death within 6 months, nonfatal myocardial infarction, stent thrombosis). No significant difference was found between moderate and severe bleeding events between the two groups [145].

The TRIGGER-PCI (Testing Platelet Reactivity In Patients Undergoing Elective Stent Placement on Clopidogrel to Guide Alternative Therapy With Prasugrel) study compared clopidogrel and prasugrel based on platelet reactivity tested with the VerifyNow assay in stable coronary artery disease patients undergoing elective PCI. Although switching from clopidogrel to prasugrel afforded effective platelet aggregation inhibition, event rate was extremely low in these patients irrespective of the response to antiplatelet therapy, not demonstrating a clinical utility of this strategy [146].

The ARCTIC trial (The Assessment by a Double Randomization of a Conventional Antiplatelet Strategy versus a Monitoring-guided Strategy for Drug-Eluting Stent Implantation and of Treatment Interruption versus Continuation One Year after Stenting) investigated a total of 2440 lower-risk cardiovascular patients who were randomized to a group where antiplatelet drug dose adjustments were performed based on the result of platelet aggregometry testing, and a conventional group without monitoring and drug dose adjustments. No significant difference was found between the two treatment arms of the study in the primary outcomes (composite death of any cause, myocardial infarction, stent thrombosis, stroke, need for urgent revascularisation within 1 year) [147].

The results of the ANTARTIC study (Assessment of a Normal Versus Tailored Dose of Prasugrel After Stenting in Patients Aged >75 Years to Reduce the Composite of Bleeding, Stent Thrombosis and Ischemic Complications) implied that monitoring platelet function and individualizing antiplatelet therapy does not improve outcomes for elderly ACS patients undergoing coronary stenting [148]. This study was performed on 877 patients aged 75 years or more who were at a very high risk of ischaemic and bleeding complications. All patients were started on prasugrel therapy. 442 patients were randomized to the conventional therapy, while 435 to platelet function monitoring at Day 14 and -when needed- to treatment adjustment. Additional monitoring was performed at Day 28 in patients who had needed treatment adjustment. The primary end point was the composite cardiovascular death, myocardial infarction, stroke, stent thrombosis, urgent revascularisation and bleeding complication at 1 year. Platelet function monitoring led to a change of therapy in 44.8% of patients who were identified as being over or undertreated, yet this strategy did not improve ischemic of safety outcomes.

In a recent study Godschalk et al. examined 113 patients who had had stent thrombosis and found that tailored antiplatelet therapy, based on platelet function testing, reduced the rate of cardiac death and/or recurrent stent thrombosis 1 year after stent thrombosis, compared with a historical cohort of patients with stent thrombosis without tailored antiplatelet therapy [149].

While the ischemic risk reduction for prasugrel versus clopidogrel was demonstrated in the early treatment period after PCI, bleeding risk becomes prominent during the chronic phase of therapy. An ongoing study based on MEA may give and answer whether platelet function-guided de-escalation of antiplatelet treatment may influence net clinical benefit [150].

Tailored antiplatelet therapy was much less investigated in other, non-cardiological patient groups. However, in a study of 266 patients with unruptured intracranial aneurysms undergoing stent-assisted coiling, 152 patients were tested preoperatively for responsiveness to aspirin and clopidogrel. Aspirin non-responsiveness was detected in 3 patients and clopidogrel resistance in 21 patients. These patients received additional doses of antiplatelet drugs. The group undergoing antiplatelet testing, and if necessary, tailoring of antiplatelet therapy, exhibited a significantly lower rate of thrombotic complications as well as a lower rate of mortality or permanent morbidity. No significant difference was found in hemorrhagic complications between the two groups [151].

## 4. Conclusions

Platelet aggregometry testing plays a role in the diagnosis of inherited platelet function disorders in the hands of hematologists.

European and American guidelines do not recommend the routine monitoring of antiplatelet therapy before or after stenting (class III, level of evidence A); however, a class IIb recommendation with level-C evidence is given for potentially high-risk situations, including suspicion of resistance to treatment or high bleeding risk. Nevertheless, the results of novel trials that failed to demonstrate any clinical improvement with monitoring-driven antiplatelet therapy seem to challenge current international guidelines.

However, the majority of the evidences concerning platelet aggregometry testing and tailored antiplatelet therapy is based on trials that were designed for patients with coronary artery disease. Evidence is lacking for neurovascular patients with TIA or stroke, neurosurgical settings, as well as for patients with peripheral artery disease treated conservatively or undergoing vascular surgery or angiological intervention. Personalized antiplatelet therapy in patients undergoing neurovascular intervention should also be more investigated. Further studies are needed to clear whether tailored therapy may contribute to better clinical outcomes in these groups.

## Figures and Tables

**Table 1:** Recommended concentration of agonists for light transmission aggregometry in different guidelines (LTA: light transmission aggregometry; PRP: platelet-rich plasma; ADP: adenosine diphosphate).

Agonist	Cattaneo et al. [46]	Hayward et al. [38]	Harrison et al. [48]		Christie et al. [47]
		Final Concentrations of the Agonists	Recommended Starting Concentrations in PRP	Range of Final Concentrations in PRP	Final Concentrations of the Agonists
Collagen	Use a low concentration of collagen, which is sufficient to cause the aggregation of normal platelets (e.g., 2 µg/mL Horm collagen). —Higher concentrations of collagen should be used if abnormal results with 2 µg/mL	A low concentration that is verified to detect impaired platelet function from aspirin and other cyclo-oxygenase 1 inhibitors is recommended Testing with a higher concentration should be performed when there is reduced maximal aggregation with the low concentration	1.25 μg/mL (type I tendon)	1.0–5.0 μg/mL	1–5 µg/mL type 1 fibrillary tipically 2 µg/mL to start
Epinephrine	5 μM Higher concentrations of epinephrine should be used if abnormal results with 5 μM	5–10 μmol/L A higher concentration of epinephrine does not have diagnostic utility and should not be included in the panel for LTA	5 μmol/L	0.5–10 μmol/L	0.5–10 μmol/L typically 5 μmol/L
ADP	2 µM Higher concentrations of ADP should be used if abnormal results with 2 µM	2.0–10 μmol/L Higher concentrations should be tested if aggregation is impaired with 2.0–2.5 μmol/L	2.5 μmol/L	0.5–20 μmol/L	0.5-10 µmol/L, typically 5 µmol/L
Ristocetin	1.2 mg/mL If platelet agglutination induced by ristocetin 1.2 mg/mL is normal, testing should be repeated using ristocetin 0.5–0.7 mg/mL If platelet agglutination induced by ristocetin 1.2 mg/mL is absent, testing should be repeated using ristocetin 2 mg/mL	Low: 0.5–0.6 mg/mL High: 1.2–1.5 mg/mL	1.2–1.5 mg/mL	1.2–1.5 and 0.5–0.7 mg/mL (single doses)	Low: ≤0.6 mg/mL High: 0.8–1.5 mg/mL
Thromboxane A2 Analogue U46619	1 µM Higher concentrations of U46619 should be used if abnormal results with 1 µM	1.0 μmol/L	-	1.0 μmol/L (single dose)	1–2 µmol/L
Arachidonic Acid	1 mM Higher concentrations of arachidonic acid should be used if abnormal results with 1 mM	0.5–1.64 mmol/L	0.5–1.0 mmol/L (single dose)	0.5–1.0 mmol/L (single dose)	single concentration between 0.5 and 1.6 mmol/L

**Table 2:** Studies investigating the association of platelet aggregometry results and ischemic events (VASP: vasodilator-stimulated phosphoprotein; LTA: light transmission aggregometry; PCI: percutaneous coronary intervention; ST: stent thrombosis; MACE: major adverse cardiac events; ACS: acute coronary syndrome; AA: arachidonic acid; DES: drug eluting stent; HRPR: high residual platelet reactivity; MEA: multiple electrode aggregometry; TIA: transient ischemic attack).

Study	*N*	Method	Patient Group	Outcome	Conclusion
Blindt et al. [113]	99	VASP LTA	PCI	stent thrombosis (ST) (6 months)	VASP was an independent predictor of ST.
Bonello et al. [114]	144	VASP	PCI	MACE (6 months)	VASP has a very high negative predictive value, it can predict postprocedural MACE.
Breet et al. [115]	1069	LTA VerifyNow PlateletWorks IMPACT-R PFA-100	Elective PCI	MACE (1 year)	PlateletWorks, LTA, VerifyNow and Innovance P2Y were significantly associated with the primary end point.
Buonamici et al. [116]	804	LTA (ADP induced platelet aggregation)	PCI+ DES implantation	stent thrombosis (6 months)	Nonresponsiveness to clopidogrel is a strong independent predictor of ST.
Cuisset et al. [117]	598	LTA (ADP induced platelet aggregation) VASP	PCI (ACS)	ST (30 days)	ST patients had greater ADP induced aggregation but only a trend toward greater platelet reactivity index.
Hochholzer et al. [118]	802	LTA (ADP induced platelet aggregation)	elective PCI	MACE (1 month)	Platelet aggregation immediately before elective stenting correlated with early outcome.
Geisler et al. [119]	1019	LTA (ADP induced platelet aggregation)	PCI	stent thrombosis (3 months)	Early but not late ST was influenced by residual platelet aggregation.
Gori et al. [120]	746	LTA (ADP, AA and collagen induced platelet aggregation)	PCI, DES	stent thrombosis, ST+death (6 months)	Dual non-responsiveness to aspirin and clopidogrel identifies patients at a very high risk of ST and death.
Migliorini et al. [121]	215	LTA (ADP induced platelet aggregation)	PCI, DES	stent thrombosis, cardiac death	HRPR after 600 mg clopidogrel is a strong marker of increased risk of cardiac death and ST.
Marcucci et al. [122]	683	VerifyNow P2Y12	PCI (ACS)	MACE (1 year)	HRPR to ADP is able to detect patients at risk of death and nonfatal MI.
Price et al. [123]	380	VerifyNow P2Y12	PCI+DES implantation	MACE (6 months)	HRPR was associated with post-discharge events.
Siller-Matula et al. [124]	416	MEA ADP VASP	PCI	stent thrombosis (6 months)	MEA predicts stent thrombosis better than the VASP phosphorylation assay.
Szapáry et al. [125]	100	LTA (ADP induced platelet aggregation)	Stroke or TIA	ischemic stroke, myocardial infarction, critical limb ischaemia (1 year)	Patients who were clopidogrel resistant at baseline had a significantly higher rate of ischemic events compared to clopidogrel responders.

**Table 3:** Studies investigating the association of platelet aggregometry and bleeding risk (PCI: percutaneous coronary intervention LTA: light transmission aggregometry ADP: adenosine diphosphate VASP: vasodilator-stimulated phosphoprotein ACS: acute coronary syndrome PRI: platelet reactivity index MEA: multiple electrode aggregometry CPA: cone-and platelet analyser ICU: intensive care unit).

Study	Patients	Methods	Association with Bleeding
Campo et al. [126]	PCI patients (*n* = 300)	VerifyNow	Low on-clopidogrel platelet reactivity was associated with bleeding events. 1 month on-clopidogrel platelet reactivity better discriminates bleeding complications than at baseline.
Chen et al. [127]	Patients treated with clopidogrel ≤6 days before CABG (*n* = 45)	LTA (ADP-induced platelet aggregation) PFA-100	<40% pre-heparin ADP-induced aggregation predicted in 92% severe bleedings requiring transfusion.
Cuisset et al. [128]	NSTEMI +PCI (*n* = 597)	VASP ADP induced platelet aggregation	Risk of TIMI major and minor bleeding was significantly higher in hyperresponders to antiplatelet therapy.
Michelson et al. [129]	ACS + PCI (*n* = 125)	VASP	Significant association of reduced VASP PRI with the occurrence of hemorrhage event
Mokhtar et al. [130]	PCI patients (*n* = 346)	VASP	VASP index was significantly higher in patients who suffered a non-CABG related TIMI bleeding compared to patients without bleeding.
Parodi et al. [131]	PCI patients treated with prasugrel and aspirin (*n* = 45)	LTA (ADP induced platelet aggregation)	Low residual platelet reactivity and female gender were independent predictors of bleeding events.
PEGASUS-PCI [132]	PCI (*n* = 416)	MEA, VASP, CPA, PFA-100	The incidence of major bleedings was numerically higher in patients with an enhanced vs. poor response to clopidogrel assessed by MEA.
Rahe-Meyer et al. [77]	cardiac surgery (*n* = 60)	MEA	Near-patient platelet aggregation may allow the identification of patients with enhanced risk of platelet concentrate transfusion, both pre-operatively and upon arrival on the ICU.
Ranucci et al. [76]	cardiac surgery, thienopyridine treatment (*n* = 87)	MEA ADP	MEA ADP test was associated with postoperative bleeding and platelet transfusion.
